# Optimizing Interfacial Adhesion and Mechanical Performance of Multimaterial Joints Fabricated by Material Extrusion

**DOI:** 10.3390/ma18163846

**Published:** 2025-08-16

**Authors:** Jakub Zatloukal, Mathieu Viry, Aleš Mizera, Pavel Stoklásek, Lukáš Miškařík, Martin Bednařík

**Affiliations:** 1 Faculty of Technology, Tomas Bata University in Zlin, Vavreckova 5669, 760 01 Zlin, Czech Republic; mbednarik@utb.cz; 2 Prusa Development a.s., 170 00 Praha, Czech Republic; 3 Faculty of Applied Informatics, Tomas Bata University in Zlin, Nad Stranemi 4511, 760 05 Zlin, Czech Republic; mizera@utb.cz (A.M.); pstoklasek@utb.cz (P.S.); l_miskarik@utb.cz (L.M.)

**Keywords:** multimaterial 3D printing, interlayer bonding, material extrusion (MEX), mechanical properties

## Abstract

Multimaterial 3D printing is transforming the landscape of additive manufacturing, enabling the production of advanced, functional parts with tailored properties for sectors like automotive, aerospace, and engineering. However, achieving strong interlayer adhesion between different polymers remains a significant challenge, limiting the mechanical reliability. This study investigates adhesion properties of widely used materials—polycarbonate (PC), acrylonitrile styrene acrylate (ASA), polylactic acid (PLA), and polyethylene terephthalate glycol (PETG)—and enhances mechanical performance of structural joints through optimized interlayer bonding techniques. Using the Material Extrusion (MEX) method, tensile testing was employed to evaluate the mechanical strength of joints by co-depositing and bonding material layers during the printing process. The results demonstrate that specific material combinations and joint design strategies, particularly increasing the interfacial contact area and applying interlayer bonding pressure, significantly enhance tensile strength. For instance, the strength of PC/PTEG composite joints increased from 15.2 MPa (standard joint) to 29.9 MPa (interlayer bonding strategy), nearly doubling the bond strength. These findings provide valuable insights into the behavior of multimaterial joints and propose practical approaches for improving the durability and functionality of 3D-printed structures. This research lays the groundwork for advancing multimaterial additive manufacturing, with implications for high-performance applications in engineering, aerospace, and beyond.

## 1. Introduction

Additive manufacturing (AM), commonly known as 3D printing, has revolutionized modern manufacturing by enabling the layer-by-layer creation of complex geometries directly from digital models. Among the diverse AM technologies, Material Extrusion (MEX) stands out for its accessibility, material versatility, and cost-effectiveness. Multimaterial 3D printing represents a significant advancement within additive manufacturing, enabling the fabrication of parts with multiple materials integrated into a single process. This capability allows for the creation of complex structures with tailored properties, combining materials with different mechanical, thermal, and chemical characteristics [1,2,3,4].

The technique is particularly advantageous in applications requiring multifunctionality, such as combining rigid and flexible components, conductive and insulating materials, or different polymers to enhance performance. The rise in multimaterial 3D printing addresses a growing demand in industries like aerospace, healthcare, and automotive, where performance optimization through material diversity is critical. However, the integration of different materials in a single print job introduces challenges, including ensuring compatibility between materials, controlling material transitions, and managing adhesion at interfaces. These challenges necessitate further exploration to unlock the full potential of multimaterial 3D printing [5,6,7].

Thermoplastics dominate as the primary materials for multimaterial 3D printing due to their versatility, ease of processing, and recyclability. Among these, PETG, PLA, ASA, and PC are widely used, each offering distinct mechanical and thermal properties. PLA is valued for its biodegradability and ease of use, making it a popular choice for prototypes and low-stress applications. PETG combines strength and flexibility, making it suitable for functional parts requiring durability and impact resistance [8,9,10]. ASA is known for its superior weather resistance, making it ideal for outdoor applications, while PC provides high impact strength and thermal stability, often used in engineering and high-performance contexts. These materials were specifically chosen for this study because they represent a diverse range of mechanical and thermal properties, enabling a comprehensive investigation into the challenges and opportunities of multimaterial 3D printing. Their widespread industrial use further highlights the relevance of optimizing their interactions in multimaterial systems, particularly for applications requiring strong, reliable interfaces [5,8,11].

The choice of materials in multimaterial 3D printing directly influences the mechanical properties of the final product. Understanding their individual behaviors and interactions in layered structures is critical for achieving optimal performance, particularly in applications that demand strong interlayer bonding and mechanical reliability. Despite their importance, significant gaps remain in understanding the mechanical properties of multimaterial joints. Most studies focus on single-material 3D printing, leaving multimaterial interfaces underexplored. This lack of focus limits the ability to optimize printed parts for applications where strength and durability of joints between materials are critical. Furthermore, although PETG, PLA, ASA, and PC are commonly used materials, systematic studies on their behavior when combined in layered structures are scarce. Previous efforts to enhance interfacial adhesion in multimaterial MEX have often focused on optimizing process parameters, modifying material formulations, or applying surface treatments. While these approaches are valuable, less systematic attention has been paid to the influence of the macro-level geometrical design of the joint itself as a primary strategy for improving bond strength between common, unmodified neat polymers like those investigated here [3,5,7,8,12,13,14].

Industrial significance: Multimaterial MEX joints enable property sets unattainable by single polymers, e.g., high joint strength with simultaneous toughness or chemical/UV resistance (PC/PTEG or PC/ASA), allowing lightweight structures with locally tailored properties (packaging, fixtures, outdoor parts) without the need for secondary assembly or bonding. Academic relevance: The study further contributes to the understanding of the adhesion mechanism in multimaterial MEX interfaces—we propose a model based on intimate contact, interface thermal history, and chain repetition/diffusion over Tg of both amorphous components, with the influence of contact pressure and viscosity mismatch. We build this framework on classical theories of polymer interface healing and on bond formation and interlayer contact/pressure models for FFF [3,15,16,17,18,19,20,21,22,23,24].

The mechanical response of MEX parts is strongly governed by process parameters (e.g., infill, quantity of perimeters, layer height, and print speed) [8,21]. To isolate the effects of material pairing and joint architecture, we employed a single, standardized print profile throughout this study and compared joint designs under otherwise identical conditions.

Accordingly, this work investigates the MEX interfacial bonding of PC, ASA, PLA, and PETG by comparing the tensile performance of three joint designs: standard butt joint, increased contact area (‘tooth’), and interlayer bonding. Mechanical testing reveals that optimized joint design significantly enhances adhesion, with the “tooth” and interlayer bonding strategies substantially increasing strength for most pairs compared to standard joints. Specifically, the PC/PTEG composite joint strength nearly doubled, reaching ~30 MPa with interlayer bonding, demonstrating the efficacy of this approach.

## 2. Materials and Methods

### 2.1. Equipment and Materials

The specimens were manufactured using the Original Prusa XL Assembled 5-toolhead 3D Printer—Enclosure Bundle (Prusa Research, Prague, Czech Republic). This 3D printer featured five independent tool heads, allowing for precise multimaterial printing within a single build. The enclosed chamber ensured consistent temperature control, which was particularly beneficial for materials prone to warping or requiring controlled thermal conditions. The printer’s specifications included a large build volume (360 mm × 360 mm × 360 mm) and compatibility with multiple filament types due to an advanced tool head swapping mechanism. The enclosed configuration also minimized external environmental influences during the printing process, ensuring reproducibility across samples. A tool offset calibration was performed using a camera, which enabled more accurate multimaterial printing with a precision of 0.1 mm.

Prusament PC Blend Urban Grey (Prusa Research, Prague, Czech Republic). Each filament had a standard diameter of 1.75 mm. Based on manufacturer recommendations and preliminary single-material tests, the following printing temperatures were used: PLA (Nozzle 225 °C, Bed 60 °C), PETG (Nozzle 250 °C, Bed 80 °C), ASA (Nozzle 260 °C, Bed 100 °C), and PC Blend (Nozzle 275 °C, Bed 100 °C). The selection of material pairs for multimaterial testing, PLA/PTEG composite (PLA/PTEG), PC/PTEG composite (PC/PTEG), ASA/PETG composite (ASA/PETG), and PC/ASA composite (PC/ASA), was primarily guided by bed temperature compatibility; pairs requiring vastly different bed temperatures (e.g., PC and PLA) were excluded due to anticipated difficulties in achieving reliable first-layer adhesion and minimizing warpage for both materials simultaneously on a shared build plate. Compromise bed temperatures were used for the tested pairs: 70 °C for PLA/PTEG, 90 °C for PC/PTEG and ASA/PETG, and 100 °C for PC/ASA.

### 2.2. Test and Parameters

To ensure consistency across all printed samples, standard printing parameters considered suitable for the Original Prusa XL printer (Prusa Research, Prague, Czech Republic) and the fabrication of mechanical testing specimens were employed. A layer height of 0.2 mm and a layer width of 0.5 mm were maintained. The infill density was set to 100% using a rectilinear pattern oriented at 0°/90° to optimize mechanical properties. The printing speed was adjusted to 150 mm/s for general printing, representing a standard operational speed for this printer model, while the first layer was printed slower at 40 mm/s to promote bed adhesion. To enhance edge definition and interlayer adhesion, two perimeters were applied.

All slicing and print preparation were conducted using PrusaSlicer version 2.8.1 (Prusa Research, Prague, Czech Republic). This software allowed precise tuning of multimaterial parameters, ensuring consistent extrusion and optimized filament transitions. The selected settings were tailored to align with the specific material properties, enabling the production of high-quality specimens for subsequent mechanical testing.

Mechanical properties were evaluated by tensile tests in accordance with ISO 527-1 [25]. Type 1A specimens were tested on a LabTest 6.50 (Labortech, Opava, Czech Republic). For each condition (material pair × joint type), we tested ten specimens (*n* = 10) to enable statistical evaluation.

**Figure 1:**
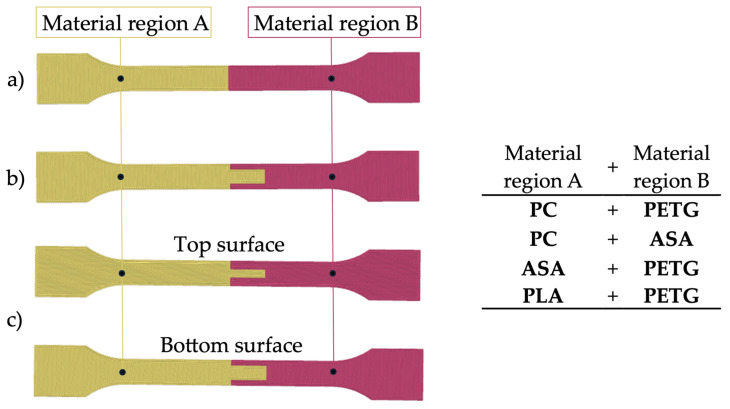
Schematic overview of the tested specimen geometries representing different joint strategies: (**a**) standard butt joint (Test 2), (**b**) joint with increased contact area (‘tooth strategy’, Test 3), and (**c**) joint with increased contact area and interlayer bonding pressure (Test 4). All the multimaterial specimens (**a**–**c**) were printed using combinations of PETG, PLA, ASA, and PC under consistent MEX conditions.

A total of four types of tests were conducted using the specified materials, as illustrated in Figure 1. Test 1 served as the reference for all subsequent outputs. Test 2 (Figure 1a) established the baseline for the material combinations. Test 3 (Figure 1b) involved a combination of materials with an increased contact area between layers. Test 4 utilized the same combination of materials with an increased contact area; however, the individual layers were pressed together to enhance interfacial contact. In Figure 1c the distinction between even and odd layers is clearly observable.

The fracture surface analysis of the tested specimens was conducted using a KEYENCE VHX-6000 digital microscope (KEYENCE, Mechelen, Belgium). The imaging process was performed at magnifications of 30× and 150×, allowing for a detailed examination of the fracture morphology. The acquired images were subjected to optical analysis, focusing on the characterization of fracture mechanisms and surface features. The evaluation was carried out following tensile testing, ensuring a comprehensive assessment of the failure characteristics of the material.

## 3. Results

### 3.1. Mechanical Properties–Reference Measurements

**Figure 2:**
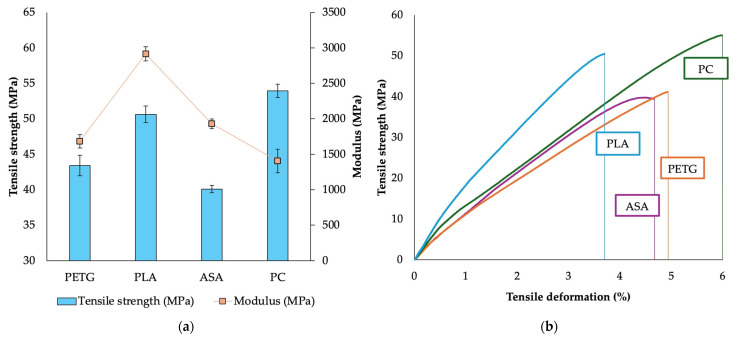
Test 1—reference measurement—tensile strength and modulus (**a**), tensile curves (**b**).

The mechanical testing revealed significant differences in the tensile properties of the tested materials (Figure 2). PC exhibited the highest tensile strength (54 MPa), followed by PLA (50 MPa), confirming their suitability for load-bearing applications. PETG (43 MPa) and ASA (40 MPa) showed lower tensile strength values.

The modulus of elasticity was highest for PLA (2915 MPa), indicating higher stiffness. This lower stiffness, typical for amorphous PC in contrast to the semi-crystalline structure of PLA [8], combines with PC’s high tensile strength to yield its notable toughness. These findings are in agreement with other publications, where they emphasize the stiffness of PLA due to its semicrystalline structure [8].

In summary, PLA is an optimal choice for rigid structural components, while PC balances high strength with ductility, making it suitable for load-bearing yet flexible applications. PETG and ASA, on the other hand, offer a well-balanced combination of strength and flexibility, making them viable options for applications requiring impact resistance and environmental durability.

### 3.2. Mechanical Properties—Composites

**Figure 3:**
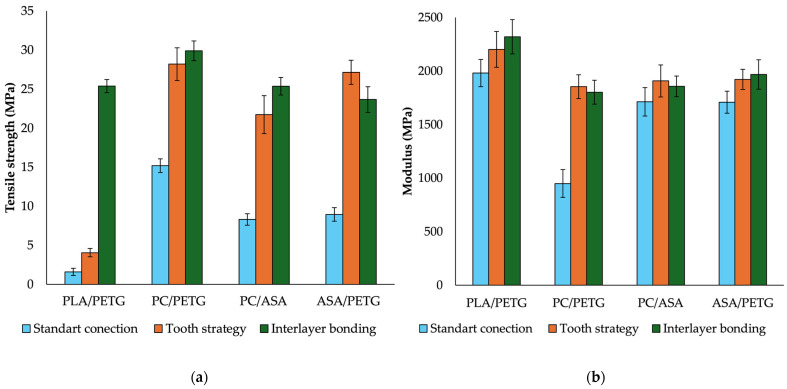
Tensile strength (**a**) and modulus (**b**) for composite samples, including Test 2—standard connection; Test 3—tooth strategy; and Test 4—interlayer bonding.

The tensile strength results (Figure 3a) reveal significant variations among the tested material combinations. The PC/PTEG blend exhibits the highest tensile strength, approximately 15.2 MPa, indicating strong mechanical performance and good interlayer adhesion between these materials. In contrast, PLA/PETG demonstrates the weakest tensile strength, measuring only 1.6 MPa, suggesting poor interfacial bonding and limited load transfer capability. The PC/ASA and ASA/PETG blends achieve intermediate tensile strength values, approximately 8.3 MPa and 8.8 MPa, respectively, signifying adequate mechanical properties but falling short of the superior performance observed in PC/PTEG.

Conversely, the elastic modulus results (Figure 3b) reveal a different trend. The PLA/PETG combination exhibits the highest modulus, approximately 2000 MPa, highlighting its rigidity despite its poor tensile strength. On the other hand, PC/PTEG, which demonstrated the highest tensile strength, displays the lowest modulus at 950 MPa, indicating lower stiffness, and ductility. The PC/ASA and ASA/PETG samples exhibit moderate modulus values, ranging from 1715 MPa to 1710 MPa, offering a balanced mechanical response in terms of stiffness and flexibility.

The tensile strength results (Figure 3a) indicate a significant improvement due to the increased contact area between materials. PC/PTEG achieves the highest tensile strength at 28.2 MPa, representing a substantial increase compared to previous values and confirming the strong compatibility of these materials. ASA/PETG follows closely with 27.1 MPa, demonstrating that this combination effectively utilizes the enlarged contact area to enhance mechanical performance. PC/ASA reaches 21.7 MPa, also showing a notable improvement. Although PLA/PETG remains the weakest combination, it exhibits a slight increase to 4 MPa, indicating some benefits from the increased contact area, albeit with limited mechanical interaction between the materials.

The elastic modulus results (Figure 3b) show less pronounced changes compared to tensile strength. PLA/PETG maintains the highest stiffness at 2200 MPa, confirming its rigid nature despite its low tensile strength. The PC/ASA and ASA/PETG combinations exhibit balanced stiffness values around 1910 MPa, indicating minor improvements and stable mechanical properties. In contrast, PC/PTEG, which achieved the highest tensile strength, has the lowest elastic modulus at 1850 MPa, indicating lower stiffness (higher compliance). The increased contact area primarily enhances tensile performance, while its influence on material stiffness remains less significant.

The tensile strength results (Figure 3a) confirm the positive impact of interlayer bonding on the mechanical performance of all the tested material combinations. PC/PTEG achieves the highest tensile strength (29.9 MPa), indicating excellent compatibility between PETG and PC, leading to strong interfacial adhesion. PLA/PTEG and PC/ASA (~25.3 MPa) show comparable values, while ASA/PETG (23.6 MPa) is slightly lower. These results highlight that mechanical properties are not solely dictated by the individual components but are significantly influenced by the quality of the interlayer interface, which is enhanced through interlayer bonding.

The modulus results (Figure 3b) show that PLA/PTEG (2320 MPa) exhibits the highest stiffness, consistent with the well-known mechanical characteristics of PLA. ASA/PETG (1970 MPa) follows closely, providing a good balance between strength and rigidity. PC/ASA (1860 MPa) and PC/PTEG (1800 MPa) display slightly lower modulus values, suggesting higher compliance compared to PLA/PTEG.

Overall, interlayer bonding plays a crucial role in improving mechanical performance, with PC/PTEG excelling in strength and PLA/PTEG demonstrating superior stiffness, making these combinations suitable for mechanically demanding applications.

**Figure 4:**
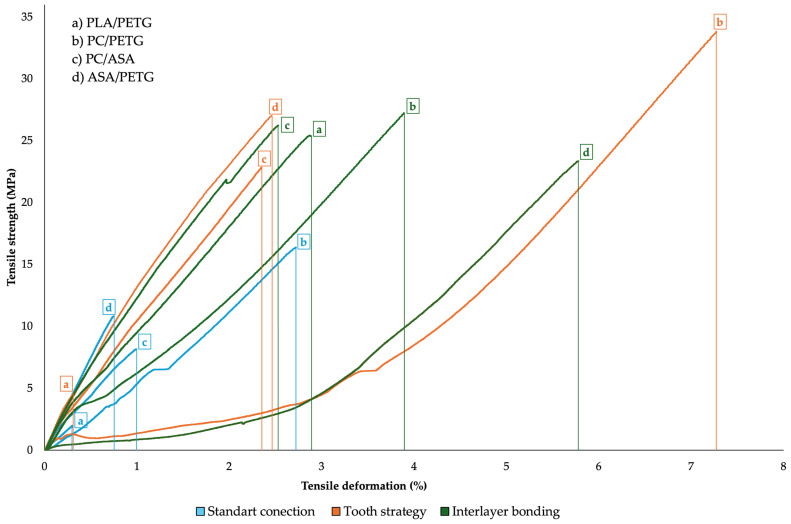
Tensile curves for composite samples: Test 2—standard connection; Test 3—tooth strategy; Test 4—interlayer bonding.

Figure 4 presents the tensile strength versus tensile deformation for four FDM material combinations under three different layer-joining strategies. The graph shows only one representative result (one tensile specimen) from the entire dataset, yet the trend clearly demonstrates the substantial influence of joining strategy on mechanical performance. The highest tensile strength (~35 MPa) and the greatest elongation (>7%) were observed for the PC/PETG combination using the tooth strategy. Interlayer bonding consistently achieved high values (approximately 23–27 MPa) across all combinations, with moderate elongation, indicating a good balance between strength and ductility. In contrast, the standard connection exhibited the lowest strength (<17 MPa) and limited elongation, confirming the restricted effectiveness of conventional layer joining. Material pairs containing PC generally showed higher elongation than those with PLA, likely due to differences in interfacial adhesion and toughness.

**Figure 5:**
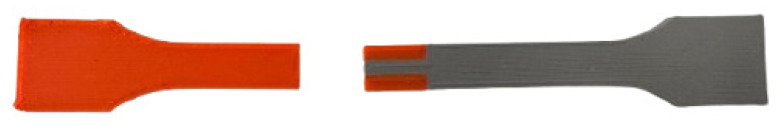
PC/PTEG specimens after tensile testing.

As previously discussed, the PC/PTEG combination exhibited the highest tensile strength among the multimaterial joints, reaching 29.9 MPa. This value is lower compared to the reference specimens printed from single materials, where PETG achieved 43 MPa and PC 54 MPa, respectively. As illustrated in Figure 5, failure occurred beyond the bonded interface, in the region where the cross-sectional area of the individual materials was reduced. This geometric discontinuity likely contributed to the lower tensile strength observed in the multimaterial specimen.

Kuipers and Su [22] previously investigated multimaterial printing involving a PLA–PP combination, employing both form-lock mechanisms and interlayer bonding strategies. Their reported tensile strength values ranged from 6 to 7 MPa. In contrast, the results obtained in this study demonstrate significantly higher interfacial strength, with most tested material combinations exhibiting tensile strengths between 20 and 30 MPa. This suggests a higher degree of mechanical compatibility among the selected thermoplastics used in our experiments.

### 3.3. Morphology

**Figure 6:**
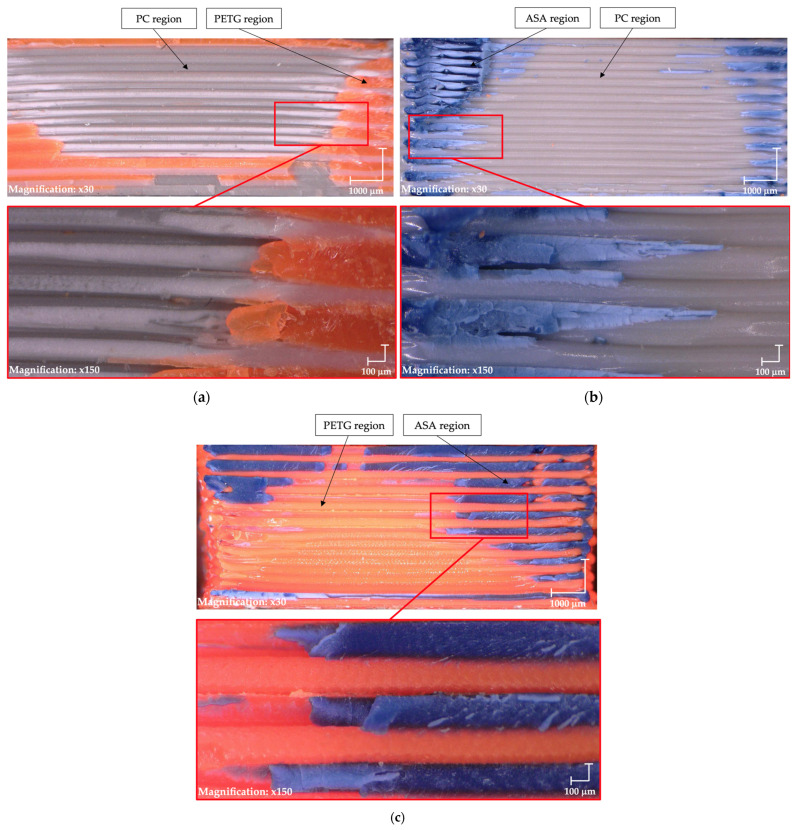
Test 2: (**a**) PC/PTEG, (**b**) PC/ASA, (**c**) ASA/PETG.

Figure 6 presents a detailed view of the fracture surfaces of the tested specimens. The PC/ASA (Figure 6a) and ASA/PETG (Figure 6b) material combinations exhibit adhesion predominantly at the outer edges of the specimens, which corresponds to their intermediate mechanical performance. In contrast, the ASA/PETG sample (Figure 6c) demonstrates adhesion not only at the edges but also within the central region of the specimen, contributing to its enhanced mechanical properties. This suggests that a more uniform interfacial bonding distribution leads to improved overall mechanical performance. This morphology is attributed to an interface temperature that remained above the glass-transition range of both polymers long enough to permit chain interdiffusion, while deposition pressure enhanced intimate wetting.

**Figure 7:**
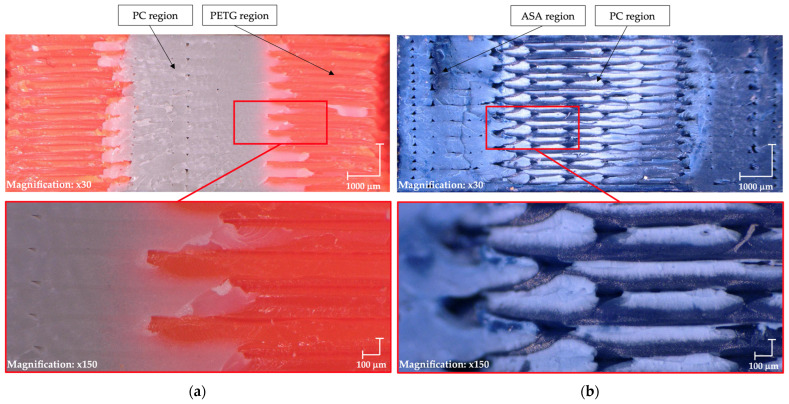

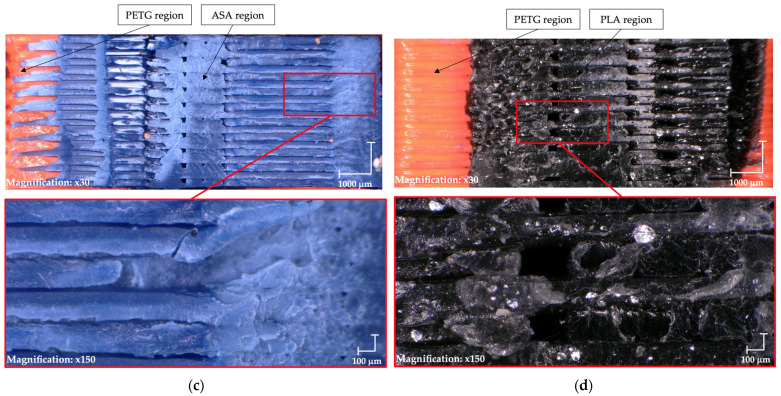
Test 3: (**a**) PC/PTEG, (**b**) PC/ASA, (**c**) ASA/PETG, (**d**) PLA/PTEG.

Figure 7a presents the PC/PTEG combination, where the materials exhibit improved bonding attributed to the increased contact area. A similar trend is observed in Figure 7b,c, further indicating that the enhanced surface interaction contributes to better adhesion between the materials.

**Figure 8:**
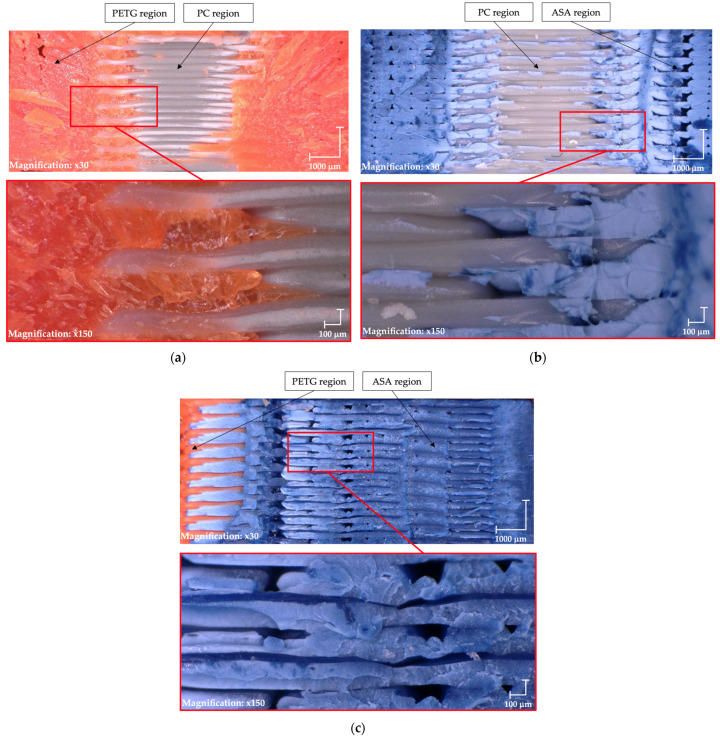
Test 4: (**a**) PC/PTEG, (**b**) PC/ASA, (**c**) ASA/PETG.

In Figure 8a, the interface between PC and PETG exhibits a distinct fusion, indicating strong interfacial adhesion, which contributes to the excellent mechanical properties of the material combination. A similar trend is observed in Figure 8b,c, further supporting the enhanced compatibility and structural integrity of the bonded materials.

## 4. Discussion

In this study, we analyze the mechanical properties and interfacial connections and explicitly compare our trends with prior reports on multimaterial MEX, which identify interfacial time–temperature history and real contact area as primary drivers of bond strength [12,21]. The selection of material combinations was based on their printability, ensuring that the printed specimens met the required quality standards for mechanical testing.

For PC–ASA, the observed rise from 8.3 MPa (butt) to 21.7 MPa (tooth) and 25.3 MPa (pressure-assisted) mirrors the literature where interlocked geometries consistently exceed simple face-to-face contacts in dissimilar pairs [26]. The additional gain under pressure aligns with interface-diffusion models and contact-pressure consolidation evidenced in MEX hot-rolling studies [27,28]. These comparisons support our interpretation that geometry primarily enables load transfer, while pressure/thermal assistance further consolidates the weld.

The PC/ASA combination is particularly suited for applications requiring high strength, impact resistance, and excellent chemical stability. PC provides superior tensile strength, while ASA contributes outstanding impact resistance and weatherability. These properties make this combination ideal for manufacturing specialized ventilation ducts or sleeves for air intake and exhaust systems, particularly in the automotive and aerospace industries, where material properties such as strength, toughness, and environmental resistance are critical. This synergy between PC and ASA enables their use in demanding environments requiring long-term performance and durability.

PETG and ASA exhibit moderate compatibility in multimaterial 3D printing, attributed to their distinct yet complementary mechanical properties. In Test 2, utilizing a single butt joint, the ASA/PETG combination achieved a tensile strength of 8.9 MPa, demonstrating better initial adhesion compared to PLA/PTEG, though inferior to PC/PETG. The implementation of the “tooth strategy” in Test 3 significantly enhanced tensile strength to 27.1 MPa, with a corresponding strain of approximately 2%. This improvement highlights the positive effect of increased contact area on mechanical interlocking and adhesion between the two materials. However, in Test 4, the application of interlayer bonding unexpectedly resulted in a reduction in tensile strength to 23.6 MPa, suggesting that this bonding strategy is not optimal for the ASA/PETG combination.

The combination of PETG and ASA offers a balance of properties suitable for specific practical applications. PETG contributes its toughness and ease of processing, while ASA provides excellent weather resistance and impact strength. This makes ASA/PETG a viable option for outdoor components, such as protective casings, covers, or structural elements exposed to variable weather conditions. Additionally, this combination could be advantageous in applications requiring a balance between mechanical performance and environmental resistance, such as custom parts in the construction or automotive industries.

The combination of PETG and PC demonstrates outstanding compatibility in multimaterial 3D printing, leveraging PETG’s toughness and PC’s high tensile strength and heat resistance.

In Test 2, employing a simple butt joint, the PC/PTEG combination achieved the highest tensile strength among the tested materials at 15.2 MPa. The implementation of the “tooth strategy” in Test 3 significantly enhanced tensile strength to 28.2 MPa, highlighting the improved mechanical load transfer enabled by the increased contact area. Further optimization in Test 4, utilizing interlayer bonding, resulted in a tensile strength of 29.9 MPa.

The PC/PTEG blend exhibits similar chemical characteristics, including comparable polarity and the presence of aromatic rings in their respective structures. Furthermore, their processing temperature windows are proximate (PETG: 230–260 °C; PC: 250–275 °C), which facilitates similar cooling rates. This thermal compatibility likely enhances chain interdiffusion and entanglement across the interlayer interface.

The combination of PETG and PC is well-suited for applications requiring high mechanical strength and resistance to temperature fluctuations. PETG contributes toughness and chemical stability, while PC adds high tensile strength and impact resistance. Specifically, achieving joint strengths near 30 MPa with optimized strategies elevates this combination beyond prototyping, making it suitable for manufacturing functional components, such as custom machinery components, durable robotic end-effectors, or high-impact electronic device housings where interface reliability is paramount. PC/PTEG provides a balance of strength, flexibility, and long-term durability, making it a versatile choice for advanced multimaterial applications PC/PTEG.

PETG and PLA exhibit substantial differences in mechanical properties, which influence their compatibility in multimaterial 3D printing applications. In Test 2, the material combination demonstrated very low tensile strength (1.6 MPa) and minimal deformation, primarily due to poor adhesion and limited interfacial contact area. The implementation of the “tooth strategy” in Test 3 led to a 2.5-fold increase in tensile strength (4 MPa); however, deformation remained low, indicating persistent interfacial fragility. A notable improvement was observed in Test 4, where interlayer bonding significantly enhanced tensile strength to 25.4 MPa, suggesting the potential applicability of this material combination in stressed environments.

In contrast, the PLA/PTEG combination presents significant structural disparities. PLA lacks aromatic rings and predominantly features a linear molecular architecture with higher polarity due to the exclusive presence of ester groups. The lower processing temperature range of PLA (180–220 °C) results in faster solidification, consequently limiting the extent of polymer chain diffusion and interfacial interaction with the PETG phase.

Despite these advancements, the PLA/PTEG combination remains less effective than other tested material pairings, primarily due to poor chemical adhesion. However, this limitation can be advantageous in specific applications, such as using one material (e.g., PLA) as a support structure and the other (e.g., PETG) as the primary material. The weak adhesion between these materials facilitates the easy removal of supports without damaging the primary structure, making PLA/PTEG a practical choice for support-material combinations in multimaterial 3D printing.

## 5. Conclusions

This study investigated the adhesion between different material combinations (PC, ASA, PLA, and PETG) and the optimization of structural joints via joint design strategies in multimaterial MEX printing. The main findings can be summarized as follows:

- The PC/PTEG combination exhibited the highest tensile strength (29.9 MPa) among the tested pairs using the interlayer bonding strategy, demonstrating excellent mechanical compatibility and strong interfacial adhesion.
- The interlayer bonding strategy (Test 4) significantly enhanced the joint strength compared to standard butt joints for the PLA/PTEG, PC/PTEG, and PC/ASA combinations.
- Conversely, the interlayer bonding strategy did not provide further improvement for the ASA/PETG combination compared to the “tooth strategy” (Test 3), indicating limitations in the bonding mechanism for this specific pair under the tested conditions.
- The results provide valuable quantitative data on the compatibility and achievable joint strength for these common MEX material pairs, aiding in material selection and interface design for multimaterial applications.
- The unexpected behavior of the ASA/PETG pair under the interlayer bonding strategy warrants further investigation into the specific adhesion mechanisms involved.

These findings contribute to advancing the development of stronger and more reliable multimaterial 3D-printed structures. Future work should focus on further optimizing joint geometries, refining interfacial bonding techniques, and potentially exploring the influence of print parameters on these optimized joints.

## Data Availability

The raw data supporting the conclusions of this article will be made available by the authors on request.
